# Biochar and Chitosan Regulate Antioxidant Defense and Methylglyoxal Detoxification Systems and Enhance Salt Tolerance in Jute (*Corchorus olitorius* L.)

**DOI:** 10.3390/antiox10122017

**Published:** 2021-12-19

**Authors:** Mirza Hasanuzzaman, Md. Rakib Hossain Raihan, Ebtihal Khojah, Bassem N. Samra, Masayuki Fujita, Kamrun Nahar

**Affiliations:** 1 Department of Agronomy, Sher-e-Bangla Agricultural University, Dhaka 1207, Bangladesh; rakib.raihan1406185@sau.edu.bd; 2 Department of Food Science and Nutrition, College of Science, Taif University, P.O. Box 11099, Taif 21944, Saudi Arabia; eykhojah@tu.edu.sa; 3 Department of Biology, College of Science, Taif University, P.O. Box 11099, Taif 21944, Saudi Arabia; b.elsayed@tu.edu.sa; 4 Laboratory of Plant Stress Responses, Faculty of Agriculture, Kagawa University, Miki-cho, Kita-gun, Kagawa 761-0795, Japan; fujita@ag.kagawa-u.ac.jp; 5 Department of Agricultural Botany, Sher-e-Bangla Agricultural University, Dhaka 1207, Bangladesh

**Keywords:** abiotic stress, salinity, fiber crop, antioxidant defense, biostimulants, organic amendments

## Abstract

We investigated the role of biochar and chitosan in mitigating salt stress in jute (*Corchorus olitorius* L. cv. O-9897) by exposing twenty-day-old seedlings to three doses of salt (50, 100, and 150 mM NaCl). Biochar was pre-mixed with the soil at 2.0 g kg^−1^ soil, and chitosan-100 was applied through irrigation at 100 mg L^−1^. Exposure to salt stress notably increased lipid peroxidation, hydrogen peroxide content, superoxide radical levels, electrolyte leakage, lipoxygenase activity, and methylglyoxal content, indicating oxidative damage in the jute plants. Consequently, the salt-stressed plants showed reduced growth, biomass accumulation, and disrupted water balance. A profound increase in proline content was observed in response to salt stress. Biochar and chitosan supplementation significantly mitigated the deleterious effects of salt stress in jute by stimulating both non-enzymatic (e.g., ascorbate and glutathione) and enzymatic (e.g., ascorbate peroxidase, dehydroascorbate reductase, monodehydroascorbate reductase, glutathione reductase superoxide dismutase, catalase, peroxidase, glutathione *S*-transferase, glutathione peroxidase) antioxidant systems and enhancing glyoxalase enzyme activities (glyoxalase I and glyoxalase II) to ameliorate reactive oxygen species damage and methylglyoxal toxicity, respectively. Biochar and chitosan supplementation increased oxidative stress tolerance and improved the growth and physiology of salt-affected jute plants, while also significantly reducing Na^+^ accumulation and ionic toxicity and decreasing the Na^+^/K^+^ ratio. These findings support a protective role of biochar and chitosan against salt-induced damage in jute plants.

## 1. Introduction

Plants face several types of environmental stresses, such as salinity, drought, waterlogging, metal/metalloid toxicity, extreme temperature, and mechanical injury during their growth period. These stresses have unfavorable effects on crop productivity and therefore threaten global food security [1]. Among many abiotic stresses, salinity is considered one of the most devastating because around 20–50% of the currently irrigated land area in arid or semi-arid regions across the world is threatened by salinization [2]. Salinity hampers many different physiological and biochemical processes in plants by ion toxicity and osmotic stress. This then accelerates the generation of reactive oxygen species (ROS) due to imbalances created in redox homeostasis, and ROS cause oxidative damage to plant tissues [3,4].

Plants under salt stress are exposed to increased concentrations of Na^+^ and Cl^−^ ions in the root zone, and this creates an imbalance in the osmotic potential of cells and reduces water uptake capacity. High amount of Na^+^ ions are also accumulated in plants under saline conditions, and this inhibits K^+^ uptake, thereby retarding several K^+^-dependent physiological processes, such as membrane equilibrium, turgor potential, enzyme activities, photosynthesis, transpiration, and protein synthesis [5,6,7]. Salt stress also triggers the generation of ROS (e.g., superoxide, O_2_^•−^; hydrogen peroxide, H_2_O_2_; and singlet oxygen, ^1^O_2_), which disrupts the equilibrium of antioxidant defense and overwhelms their capacity to detoxify ROS. Ultimately, the excess generation of ROS causes oxidative damage and death of the plant [4]. Plants also accumulate methylglyoxal (MG), another cytotoxic compound, in response to salt stress, and this leads to further cellular dysfunction. Thus, the survival of plants under salinity stress largely depends on their ability to preserve the antioxidant and glyoxalase systems that mitigate the increases in levels of ROS and MG, respectively [8].

The plant antioxidant defense system consist of non-enzymatic antioxidants, such as ascorbate (AsA), glutathione (GSH), tocopherols, flavonoids, and carotenoids, and enzymatic antioxidants, such as ascorbate peroxidase (APX), monodehydroascorbate reductase (MDHAR), dehydroascorbate reductase (DHAR), glutathione reductase (GR), superoxide dismutase (SOD), catalase (CAT), peroxidase (POD), glutathione *S*-transferase (GST), and glutathione peroxidase (GPX), which aid in scavenging the ROS [1]. Methylglyoxal toxicity is combated by the action of two glyoxalase enzymes, glyoxalase I (Gly I) and glyoxalase II (Gly II) [8]. Plants are also equipped with ion exclusion machinery in different cellular compartments, so ion exclusion also functions to reduce the adverse impact of toxic ions in plants [9,10]. Recently, several types of organic amendments, such as biochar, chitosan, and vermicompost, have been used to prevent salt-induced damage in plants [11,12].

Biochar (BC) is a pyrolyzed product of various agricultural farm residues (i.e., stover, husks, livestock and poultry wastes, forest wastes, etc.) and contains profound amount of organic carbon, along with calcium, magnesium, and organic carbonates [13,14]. Biochar improves the soil water-holding capacity, microbial activity, permeability, carbon sequestration capability, and porosity, so it enhances plant growth and leads to higher yields [15]. BC also has the capability to bind Na^+^ ions on its exchange sites, while also increasing the soil K^+^ ion availability to maintain a consistent Na^+^/K^+^ ionic balance and help to reduce Na^+^ uptake in plants [16].

Chitosan (CHT) is a linear polysaccharide produced through deacetylation of the chitin component of the exoskeleton of several crustaceans [17]. CHT is primarily a polymer constructed from two monomers, 2-glucosamine and N-acetyl-2-glucosamine. Chitosan has positively charged ions that provide it with the capability to bind with negatively charged lipids, macromolecules, and metal ions [18]. Chitosan is nontoxic and biodegradable and shows some unique properties, such as anti-bacterial activity and hydrophilicity. It is widely used in different processes, ranging from water treatment to food packaging [11]. It is also capable of scavenging ROS, and it can improve the plant antioxidant status and soil enzyme activities [18,19]. Both BC and CHT can also induce the accumulation of osmolytes, such as proline (Pro) and glycine betaine, to maintain plant cell osmotic potential and stomatal conductance under stress, thereby improving photosynthetic activity and the net carbon assimilation rate in plants [12,20].

One plant that is adversely affected by salt stress is jute (*Corchorus olitorius* L.), a crop plant that is widely cultivated on the Asian and African continents for its bast fibers, which are used in a wide range of marketable products [21]. However, environmental stresses, such as salt stress, adversely affect both the quantity of the fibers produced by the plant and the fiber quality, as well as jute germination, growth, and physiology [22,23]. The aim of the present study was to investigate the effects of soil supplementation with BC and CHT, focusing on the mediation of ROS-induced damage due to salt stress by stimulation of the antioxidative and glyoxalase pathways in jute plants.

## 2. Materials and Methods

### 2.1. Plant Materials, Experimental Conditions, and Stress Treatments

Mature, healthy seeds of jute (*C. olitorius* L. cv. O-9897) were collected from Bangladesh Jute Research Institute and sterilized. Sun-dried soil was mixed with organic manure, urea, muriate of potash, triple superphosphate, zinc sulfate, and gypsum, according to Islam [24]. Approximately 13.5 kg of this well-prepared soil was placed into 14 L plastic pots (35 cm diameter). The texture of the experimental soil was clay loam containing 30% sand, 40% silt, and 30% clay with organic carbon 0.78% and organic matter 1.35%, having a pH of 5.7. After that, BC (made from sawdust) was thoroughly incorporated with the soil at 2 g kg^−1^ soil 7 days before seed sowing in each pot. At 20 days after sowing (DAS), one set of plants was irrigated with various doses of salt (50, 100, and 150 mM NaCl) and always maintained field capacity by irrigating with 500 mL of water or salt solution at each 5-day interval. For CHT application, a concentrated solution of chitosan-100 (Wako, Japan) was prepared by dissolving in 1% acetic acid and pH of the solution was neutral. Then, at 20 and 30 DAS during irrigation, the plant with either water or salt solution, CHT was applied at 100 mg L^−1^ solution, but not applied in the BC-amended soil. At 40 DAS, several morphological, physiological, and biochemical parameters were measured. The treatments were replicated three times.

### 2.2. Determination of Shoot and Root Length

Shoot length was measured with a measuring scale from the plant base to the uppermost leaf tip. Root length was also measured from the base to the longest branch of the root. The data were presented as the average shoot and root lengths from five randomly selected plants.

### 2.3. Measurement of Stem Diameter

Stem diameter was measured from three different points of five randomly selected plants by using a slide caliper. Then, the average value was expressed as millimeter (mm).

### 2.4. Estimation of Fresh Weight and Dry Weight

Five randomly selected plants from each treatment were uprooted and washed with distilled water (DH_2_O) to remove the adhering soil with roots. After that, roots were separated from shoots, and the fresh weight (FW) of roots and shoots was measured by a digital balance, then oven-dried at 70 °C for 72 h and weighed again for dry weight (DW). Finally, the weights were averaged and expressed as g plant^−1^.

### 2.5. Estimation of SPAD Value

Five fully expanded leaves of each treatment were randomly selected, and soil and plant analysis development (SPAD) reading was taken to measure the total chlorophyll (Chl) content by using a SPAD meter (FT Green LLC, Wilmington, DE, USA). Values of each treatment were averaged for SPAD value.

### 2.6. Determination of Relative Water Content

Using the formula of Barrs and Weatherly [25], leaf relative water content (RWC) was estimated. For this purpose, leaf lamina was plucked and weighted for the FW and immersed in DH_2_O in Petri dish for 24 h by covering it with filter paper and kept in dark. After removing the adhering excess water, the leaf lamina was weighted again for the turgid weight (TW). These leaf laminas were dried in an electric oven for 48 h by maintaining 70 °C and weighted for DW. Finally, RWC was estimated following the equation:

(1) $$\mathrm{RWC}\;\left(\%\right)=\frac{\mathrm{FW}-\mathrm{DW}}{\mathrm{TW}-\mathrm{DW}}\times 100$$

### 2.7. Determination of Proline Content

Following the method of Bates et al. [26], 0.5 g fresh leaf sample was used for estimating the Pro content, which was homogenized by adding 3% sulfosalicylic acid and centrifuged at 11,500× *g* for 12 min. Then the supernatant was heated in a water bath for 60 min at 100 °C after mixing with acid ninhydrin and glacial acetic acid. After that, the mixture was cooled down at room temperature and toluene was added and vortexed, and then the upper chromophore was subjected to spectrophotometer at 520 nm. The absorbance reading was finally plotted in a standard curve for Pro determination.

### 2.8. Estimation of Malondialdehyde Content

For estimating malondialdehyde (MDA) content, 0.5 g leaf sample was homogenized into 5% trichloroacetic acid (TCA) and centrifuged (11,500× *g*, 15 min). Then the supernatant was mixed with thiobarbituric (TBA) acid reagent containing 0.5% TBA and 20% TCA and heated for 30 min at 95 °C. After cooling, absorbance was recorded at 532 nm and non-specific absorbance was recorded at 600 nm, and finally, calculation was done by deducting the values of non-specific absorbance and an extinction coefficient of 155 mM^−1^ cm^−1^ was also used [27].

### 2.9. Determination of Hydrogen Peroxide Content

Leaf sample (0.5 g) was homogenized in TCA and followed by centrifugation. Then the aliquot was incubated for 1 h after adding potassium-phosphate (K-P) buffer (10 mM, pH 7.0) and potassium iodide (1 mM). Then the absorbance was measured by using a spectrophotometer at 390 nm [28].

### 2.10. Measurement of Electrolyte Leakage

Leaf electrolyte leakage (EL) was measured by following the method of Dionisio-Sese and Tobita [29]. Leaf sample (0.5 g) was cut into small pieces and then put into falcon tubes containing 15 mL DH_2_O. After that, the tubes were heated into a water bath at 40 °C for 1 h, and then the electrical conductivity (EC1) was measured by using electrical conductivity meter (HI-993310, Hanna, Smithfield, RI, USA) cooling up to room temperature. Then again, the falcon tubes were heated at 121 °C for 40 min by using autoclave and after cooling, final electrical conductivity (EC2) was measured. Then, EL was calculated as follows:

(2) $$\mathrm{EL}\;\left(\%\right)=\frac{\mathrm{EC}1}{\mathrm{EC}2}\times 100$$

### 2.11. Histochemical Detection of Hydrogen Peroxide and Superoxide Anion

For detecting H_2_O_2_ and O_2_^•−^, 0.1% solution of 3,3′ diaminobenzidine (DAB) and nitro blue tetrazolium chloride (NBT) was used maintaining a pH of 3.8. After incubating for 72 h in dark, leaves were thoroughly cleaned with boiling 70% ethanol to remove the solution and then a picture was taken using a digital camera [30].

### 2.12. Measurement of Ascorbate and Glutathione Content

An amount of 0.5 g leaf sample was homogenized in 5% metaphosphoric acid containing 1 mM ethylenediaminetetraacetic acid (EDTA) followed by centrifugation. Neutralization of aliquot was done by using 0.5 M K-P buffer (pH 7.0) and 0.1 M dithiothreitol (DTT) for determining total AsA and DH_2_O instead of DTT used for AsA, and absorbance was taken at 265 nm. A standard curve was used to calculate the total AsA and AsA, and dehydroascorbate (DHA) content was estimated by subtracting the values of AsA from total AsA [31].

Following the method of Hasanuzzaman et al. [32], glutathione was determined. For reduced glutathione (GSH), supernatant was neutralized by 0.5 M K-P buffer (pH 7.0) and oxidized by adding 5,5-dithio-bis(2-nitrobenzoic acid) (DTNB) and reduced with nicotinamide adenine dinucleotide phosphate (NADPH) in the presence of glutathione reductase (GR). The absorbance was measured at 412 nm by spectrophotometer. In case of oxidized glutathione (GSSG), 2-vinylpyridine was used to neutralize the extract along with K-P buffer. By using a standard curve, contents of GSH and GSSG were estimated, and GSH content was determined by subtracting the values of GSSG from total GSH.

### 2.13. Extraction of Enzyme and Determination of Free Protein

For enzyme extraction, 0.5 g leaf sample was homogenized in an ice-cooled mortar pestle by using extraction buffer containing 1 mM L-ascorbic acid (AsA), 100 mM KCl, 100 mM 0.5 M K-P buffer (pH 7.0), 5.0 mM β-mercaptoethanol, and 10% (*w/v*) glycerol following the method of Hasanuzzaman et al. [32]. Protein concentrations were measured (absorbance at 595 nm) from this extracted aliquot by following the method of Bradford [33], where a standard curve was prepared by using bovine serum albumin (BSA).

### 2.14. Estimation of Antioxidant Enzyme Activities

Following the procedure of Doderer et al. [34], lipoxygenase (LOX; EC: 1.13.11.12) activity was assayed, where linoleic acid substrate containing Tween-20 and K-P buffer (pH 6.5) was used for detecting the activity at 234 nm, and 25 mM^−1^ cm^−1^ was used as extinction coefficient for calculation.

Ascorbate peroxidase (APX; EC: 1.11.1.11) activity was estimated by using an assay buffer containing 15 mM K-P buffer (pH 7.0), 0.5 mM AsA, 0.1 mM EDTA, H_2_O_2_, and enzyme, and absorbance was measured at 290 nm. The activity was finally calculated by using the extinction coefficient of 2.8 mM^−1^ cm^−1^ [35].

For monodehydroascorbate reductase (MDHAR; EC: 1.6.5.4) activity, 50 mM Tris-HCl (pH 7.5), 2.5 mM AsA, 0.2 mM NADPH, ascorbate oxidized (AO), and enzyme was assayed at 340 nm in spectrophotometer, and calculation was done by using an extinction coefficient (6.2 mM^−1^ cm^−1^) [36].

Dehydroascorbate reductase (DHAR; EC: 1.8.5.1) was estimated according to Nakano and Asada [35] in which an assay buffer was prepared by mixing 50 mM K-P buffer (pH 7.0), 2.5 mM GSH, 0.1 mM EDTA, and DHA. The activity was recorded at 265 nm and the calculation was done by using an extinction coefficient 14 mM^−1^ cm^−1^.

Glutathione reductase (GR; EC: 1.6.4.2) activity was assayed by a reaction buffer comprising K-P buffer (pH 7.0), EDTA, GSSG, NADPH, and enzyme. The absorbance was measured at 340 nm and the value was estimated by 6.2 mM^−1^ cm^−1^ as extinction coefficient [32].

Superoxide dismutase (SOD; EC: 1.15.1.1) activity was measured by following the method of El-Shabrawi et al. [37]. Here, K-P buffer (pH 7.0), NBT, xanthine, xanthine oxidase, and enzyme were mixed together for the reaction and spectrophotometrically observed at 560 nm.

Catalase (CAT; EC: 1.11.1.6) activity was measured by degrading the enzymes with H_2_O_2_ (15 mM) and K-P buffer (50 mM, pH 7.0) following Hasanuzzaman et al. [32]. Absorbance was recorded at 240 nm, and computation was done by using extinction coefficient of 39.4 M^−1^ cm^−1^.

Peroxidase (POD; EC: 1.11.1.7) activity was measured by oxidation of guaiacol in presence of K-P buffer and H_2_O_2_, and the activity was observed at 470 nm. Extinction coefficient of 26.6 mM^−1^ cm^−1^ was used for calculation [38].

Glutathione *S*-transferase (GST; EC: 2.5.1.18) activity was assayed by a buffer comprising K-P buffer (0.25 M, pH 6.5), GSH (1.5 mM), and 1-chloro-2,4-dinitrobenzene (CDNB; 1 mM). The activity was observed at 340 nm and an extinction coefficient (9.6 mM^−1^ cm^−1^) was used for computing the activity [32].

Glutathione peroxidase (GPX; 1.11.1.9) activity was estimated by following the method of Elia et al. (2003) with slight modifications from Hasanuzzaman et al. [32]. Assay buffer containing K-P buffer (100 mM, pH 7.0), EDTA (1 mM), sodium azide (NaN_3_, 1 mM), NADPH (0.12 mM), GSH (2 mM), GR (1 unit), and H_2_O_2_ (10.50 mM) with enzyme was spectrophotometrically observed at 340 nm. For computation of the activity, an extinction coefficient (6.62 mM^−1^ cm^−1^) was used.

Glyoxalase I (Gly I; EC: 4.4.1.5) activity was assayed by a buffer which contained K-P buffer (pH 7.0), magnesium phosphate (MgSO_4_; 16 mM), GSH (100 mM), and MG (35 mM) and observed at 240 nm. For calculating the activity, 3.37 mM^−1^ cm^−1^ was used as the extinction coefficient [32].

According to the procedure of Principato et al. [39], Glyoxalase II (Gly II; EC: 3.1.2.6) activity was estimated. Here, Tris-HCl (pH 7.2), DTNB, *S*-D-lactoylglutathione (SLG), and enzymes were spectrophotometrically observed at 412 nm, and calculation was done by using 13.6 mM^−1^ cm^−1^ as an extinction coefficient.

### 2.15. Estimation of Methylglyoxal (MG) Content

According to Wild et al. [40], plant sample (0.25 g) was extracted by using 5% perchloric acid and centrifuged for 12 min at 11,500× *g*. Then the extracted sample was again centrifuged with activated charcoal and neutralized by using saturated sodium carbonate. After that, neutralized sample was mixed with sodium-phosphate (Na-P) buffer and *N*-acetyl-*L*-cysteine and kept for 15 min to form N-α-acetyl-*S*-(1-hydroxy-2-oxoprop-1-yl) cysteine which was observed spectrophotometrically at 288 nm. Finally, MG content was calculated by using a standard curve.

### 2.16. Measurement of Na^+^ and K^+^ Content

Leaf Na^+^ and K^+^ contents were measured with a Na^+^ meter (Horiba-731, Japan) and K^+^ meter (Horiba-722, Horiba, Kyoto, Japan), respectively. A fresh leaf was washed with distilled water and then squeezed to extract the sap. The sap was applied directly to the sensor of the ion meter, and data values were expressed as ppm.

### 2.17. Statistical Analysis

Statistical analyses were done using CoStat v.6.400 computer-based software (CoHort Software, Monterey, CA, USA) [41]. The means were compared using Tukey’s HSD test, and *p* ≤ 0.05 was considered statistically significant.

## 3. Results

### 3.1. Root, Length, Shoot Length and Stem Diameter

**Figure 1:**
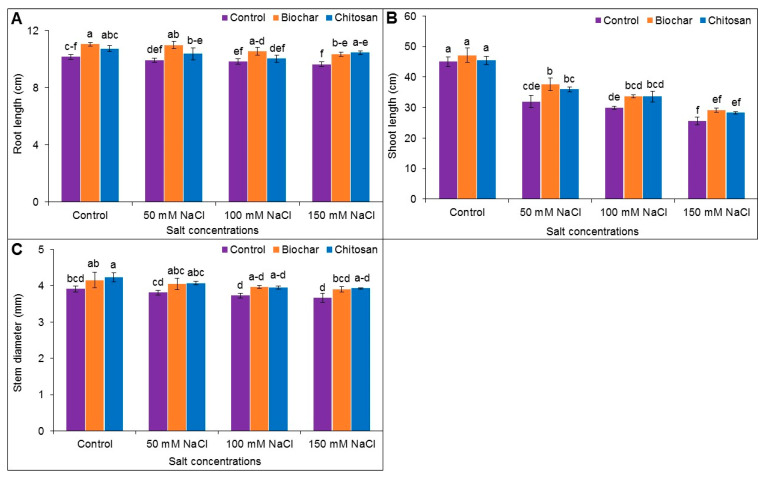
Root length (**A**), shoot length (**B**), and stem diameter (**C**) of jute as affected by biochar and chitosan under different concentrations of NaCl. Mean value (±SD) was calculated from three replications, and different letters on the columns indicate significant differences among the treatments at *p* ≤ 0.05 applying Fisher’s LSD test.

Reduction of root length was noticed in the salt-stressed plant, in comparison to untreated control (without salt and BC or CHT amendment). Amendment with BC has improved root length in salt-treated plants, and the highest increase of root length was observed at 50 mM NaCl treated plants by 10% compared to the corresponding salt-stressed plants only. On the other hand, CHT supplementation increased root length by 8% in 150 mM NaCl stressed plant, in comparison to the salt-treated plants alone (Figure 1A).

Shoot length was reduced by 41, 51, and 76% upon exposure to 50, 100, and 150 mM NaCl, respectively, in comparison to the untreated control (without salt and BC or CHT amendment). Under 50 mM NaCl-stressed plants, BC application improved the shoot length by 15%, whereas it was improved by 8% with CHT supplementation in comparison to the respective salt-stressed plant alone (Figure 1B).

The stem diameter of salt-treated plant with or without BC and CHT supplementation showed a statistically similar result compared to untreated control (without salt and BC or CHT amendment) (Figure 1C).

### 3.2. Fresh Weight (FW) and Dry Weight (DW) of Roots and Shoots

**Figure 2:**
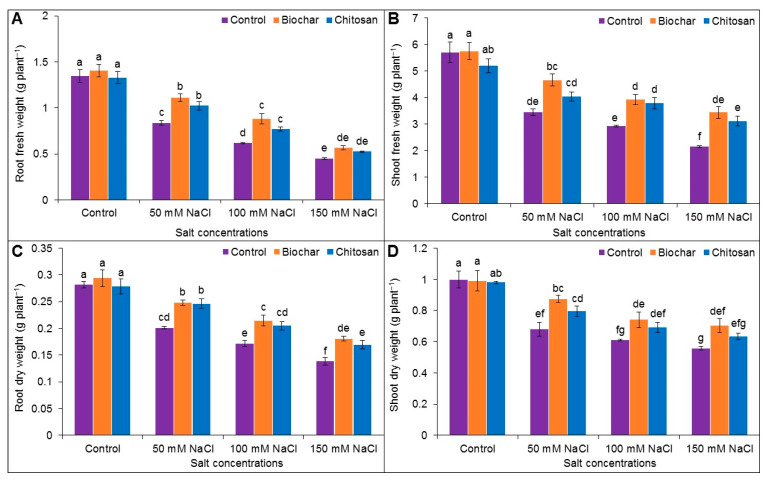
Fresh weight (**A**,**B**) and dry weight (**C**,**D**) of root and shoot of jute as affected by biochar and chitosan under different concentrations of NaCl. Mean value (±SD) was calculated from three replications, and different letters on the columns indicate significant differences among the treatments at *p ≤* 0.05 applying Fisher’s LSD test.

Exposure to 50, 100, and 150 mM NaCl reduced the root FW by 61, 119, and 201%, respectively, and reduced the shoot FW by 65, 95, and 166%, respectively, compared to the unstressed control plants. Supplementation with BC and CHT improved the root FW when compared to the corresponding salt-stressed but unsupplemented plants (Figure 2A,B).

Exposure to 50, 100, and 150 mM NaCl stress decreased the root DW by 40, 64, and 104%, respectively, and the shoot DW by 47, 64, and 79%, respectively, compared to the unstressed control plants. Supplementation with BC improved the DW of the salt-stressed plants when compared to the corresponding salt-stressed but unsupplemented plants. CHT application enhanced the root DW by 18%, 16%, and 18% in plants treated with 50, 100, and 150 mM NaCl, respectively, when compared to the corresponding salt-stressed but unsupplemented plants. CHT amendment also improved the shoot DW (by 15%) in plants exposed to 75 mM NaCl compared to salt treatment alone (Figure 2C,D).

### 3.3. Relative Water Content, Proline Accumulation, and SPAD Value

**Figure 3:**
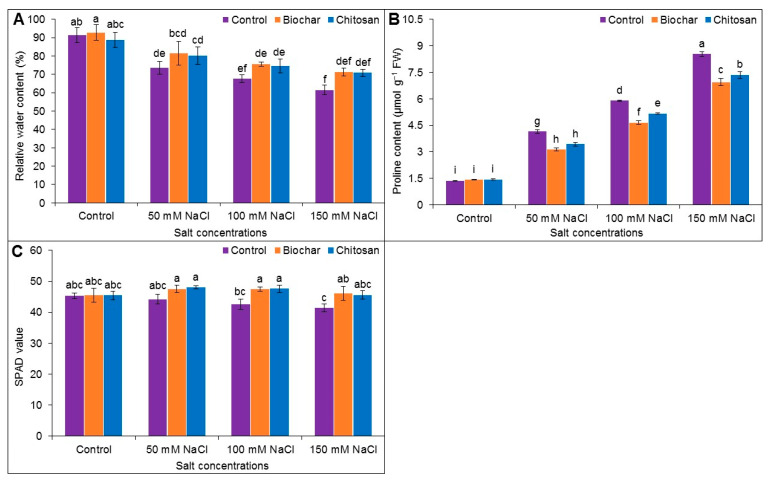
Leaf relative water content (**A**), proline content (**B**), and SPAD value (**C**) of jute as affected by biochar and chitosan under different concentrations of NaCl. Mean value (±SD) was calculated from three replications, and different letters on the columns indicate significant differences among the treatments at *p* ≤ 0.05 applying Fisher’s LSD test.

Leaf RWC declined in salt-stressed plants compared to the control plants. Exposure to 50, 100, and 150 mM NaCl reduced the RWC by 18, 24, and 30%, respectively, in comparison to unstressed control plants. When compared to the corresponding salt-stressed but unsupplemented plants, supplementation with BC increased the RWC by 8%, 8%, and 10% in plants treated with 50, 100, and 150 mM NaCl, respectively, whereas CHT supplementation improved RWC by 6%, 7%, and 9%, respectively (Figure 3A).

A sharp increase in Pro content was observed at the different levels of NaCl stress. Proline content notably increased by 67%, 77%, and 84% by 50, 100, and 150 mM NaCl, respectively, when compared to the untreated control plants. When compared to the corresponding salt-stressed but unsupplemented plants, BC supplementation reduced the Pro content by 33, 27, and 23% in plants treated with 50, 100, and 150 mM NaCl, respectively, while CHT supplementation decreased the Pro content by 21%, 14%, and 16%, respectively (Figure 3B).

The greatest reduction in the SPAD value (by 10%) was observed in plants treated with 150 mM NaCl compared to the untreated control. When compared to the corresponding salt-stressed but unsupplemented plants, BC supplementation of plants treated with 100 mM and 150 mM NaCl increased the SPAD value by 10% in both cases, and supplementation with CHT increased the SPAD value by 10% in the plants treated with 100 mM salt (Figure 3C).

### 3.4. Indicators of Oxidative Stress

**Figure 4:**
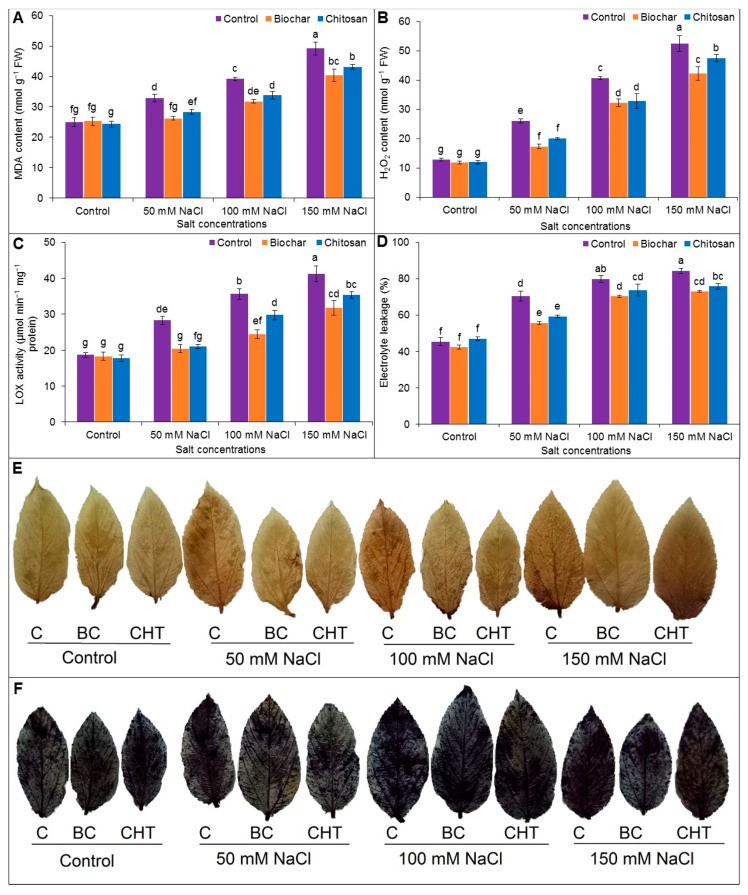
MDA content (**A**), H_2_O_2_ content (**B**), LOX activity (**C**), EL (**D**), and histochemical detection of H_2_O_2_ (**E**) and O_2_^•−^ (**F**) of jute as affected by biochar and chitosan under different concentrations of NaCl. Mean value (±SD) was calculated from three replications, and different letters on the columns indicate significant differences among the treatments at *p* ≤ 0.05 applying Fisher’s LSD test.

Exposure to salt stress significantly and dose-dependently increased the contents of MDA and H_2_O_2_, LOX activity, and EL (%) in jute leaves. The MDA content increased by 24%, 36%, and 49% in plants exposed to 50, 100, and 150 mM NaCl, respectively, compared to the untreated control plants (Figure 4A). The H_2_O_2_ content increased by 51%, 68%, and 76% in plants exposed to 50, 100, and 150 mM NaCl, respectively, compared to the untreated control plants (Figure 4B). The LOX activity increased by 34%, 48%, and 55% in plants exposed to 50, 100, and 150 mM NaCl compared to the untreated control plants (Figure 4C).

The increases in the EL (%) in jute leaves due to salt stress indicated membrane damage. The EL increased by 25%, 34%, and 39%, respectively, following exposure of plants to 50, 100, and 150 mM, respectively, compared to the untreated control plants (Figure 4D). When compared to the corresponding salt-stressed but unsupplemented plants, supplementation with BC or CHT decreased the MDA and H_2_O_2_ levels, LOX activity, and EL (%).

Salt stress caused increasing oxidative damage with increasing NaCl concentrations. Histochemical evaluation indicated a higher accumulation of H_2_O_2_ (brown spots) and O_2_^•−^ (dark blue spots) in salt-stressed jute leaves, and the spots were reduced in size and number in the salt-stressed plants supplemented with BC and CHT (Figure 4E,F).

### 3.5. Ascorbate-Glutathione Pool

**Figure 5:**
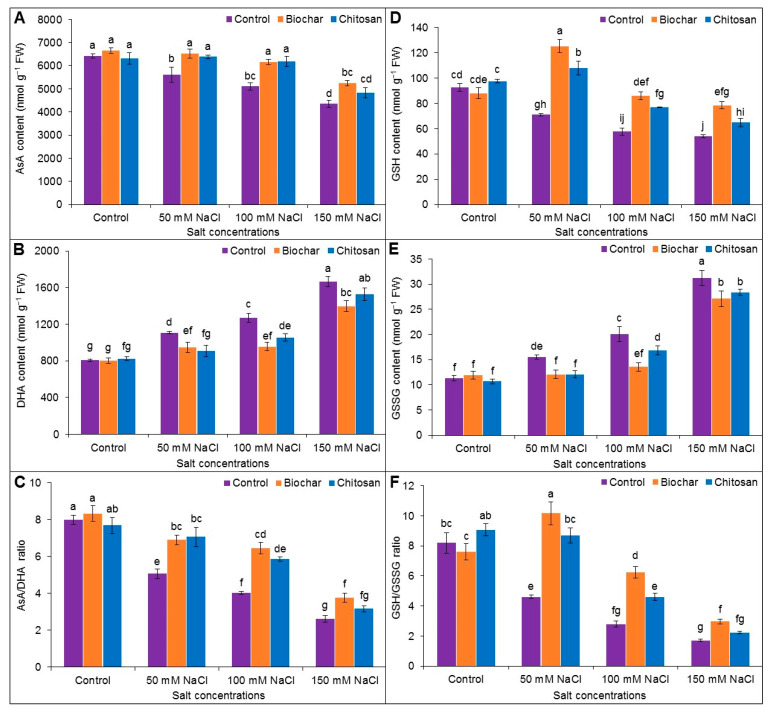
AsA content (**A**), DHA content (**B**), AsA/DHA ratio (**C**), GSH content (**D**), GSSG content (**E**), and GSH/GSSG ratio (**F**) of jute as affected by biochar and chitosan under different concentrations of NaCl. Mean value (±SD) was calculated from three replications, and different letters on the columns indicate significant differences among the treatments at *p* ≤ 0.05 applying Fisher’s LSD test.

Exposure to NaCl decreased the contents of AsA and GSH but increased the DHA and GSSG contents compared to the untreated control plants, resulting in a strong decrease in the GSH/GSSG and AsA/DHA ratios. Supplementation with BC and CHT improved the AsA/DHA ratio at all levels of NaCl stress, while CHT supplementation improved the AsA/DHA ratio in plants treated with 150 mM NaCl compared with the corresponding salt-stressed but unsupplemented treatment (Figure 5A–C). A similar trend was recorded for the GSH/GSSG ratio (Figure 5D–F). These changes in AsA/DHA and GSH/GSSG ratios were attributed to the increase in AsA and decrease in DHA, as well as the increase in GSH and decrease in GSSG level, by BC and CHT supplementation.

### 3.6. Antioxidant Enzyme Activities

**Figure 6:**
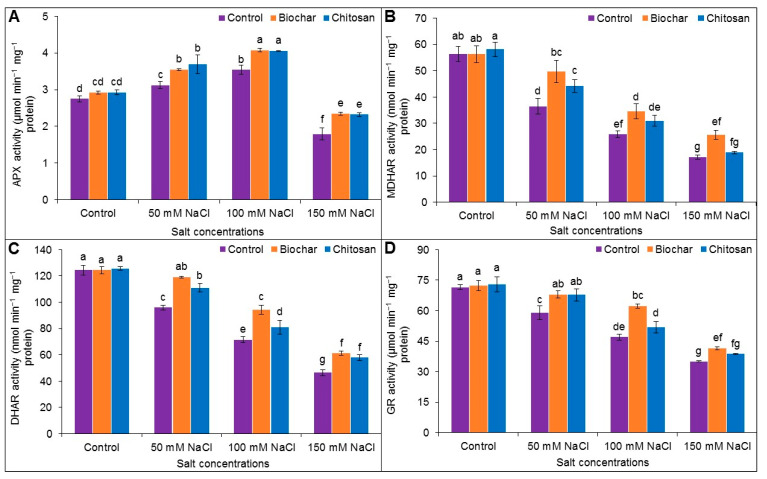
Activities of APX (**A**), MDHAR (**B**), DHAR (**C**), and GR (**D**) of jute as affected by biochar and chitosan under different concentrations of NaCl. Mean value (±SD) was calculated from three replications, and different letters on the columns indicate significant differences among the treatments at *p* ≤ 0.05 applying Fisher’s LSD test.

The APX activity increased in response to salt stress at 50 and 100 mM but not at 150 mM NaCl. The activity of MDHAR, DHAR, and GR decreased under all levels of salt stress. Supplementation with BC and CHT increased the APX activity compared to the corresponding salt-treated but unsupplemented plants. BC supplementation increased the MDHAR and GR activity at all salt levels compared to the corresponding salt-stressed but unsupplemented plants. CHT supplementation only increased MDHAR activity in plants treated with 50 mM salt stress; the differences observed for the 100 and 150 mM salt stress were not statistically significant compared to the corresponding salt-stressed but unsupplemented plants. The DHAR activity increased for all salt stress levels with BC and CHT supplementation compared to the corresponding salt-stressed but unsupplemented plants (Figure 6A–D).

**Figure 7:**
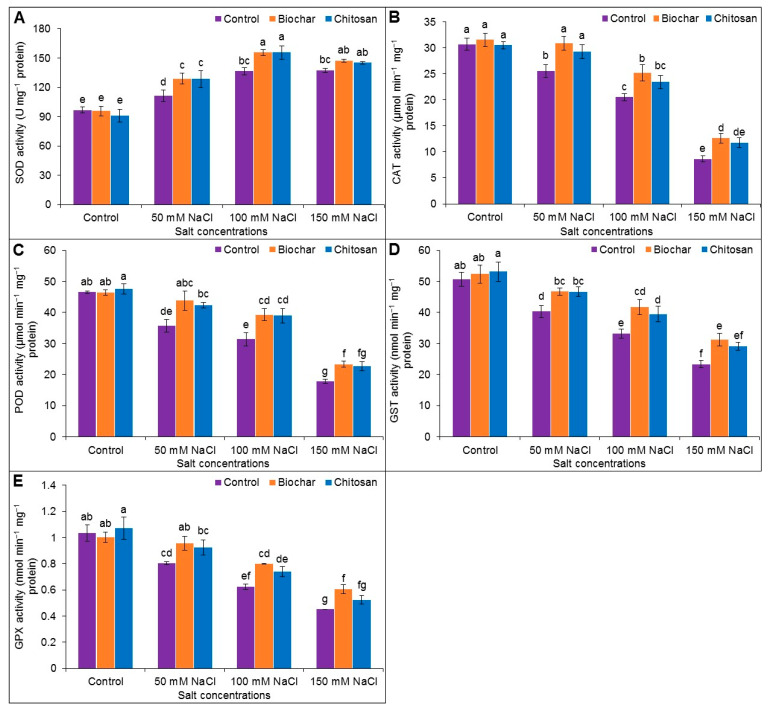
Activities of SOD (**A**), CAT (**B**), POD (**C**), GST (**D**), and GPX (**E**) of jute as affected by biochar and chitosan under different concentrations of NaCl. Mean value (±SD) was calculated from three replications, and different letters on the columns indicate significant differences among the treatments at *p* ≤ 0.05 applying Fisher’s LSD test.

Salt stress reduced CAT activity (by 20%, 50%, and 256%), POD activity (by 30%, 49%, and 163%), GST activity (by 26%, 53%, and 117%), and GPX activity (by 29%, 65%, and 129%), whereas SOD activity increased (by 13%, 29%, and 30%) in plants exposed to 50, 100, and 150 mM NaCl stress, respectively, compared to the untreated control plants. The highest SOD activity was observed with BC supplementation (by 13% and 12%) and CHT supplementation (by 13% and 12%) in plants treated with 50 and 100 mM NaCl, respectively, compared to the corresponding salt-stressed but unsupplemented plants. The SOD activity was statistically similar following BC or CHT supplementation in plants exposed to 150 mM NaCl. BC supplementation improved the CAT activity by 17%, 19%, and 32% in plants exposed to 50, 100, and 150 mM NaCl, respectively, and CHT significantly increased the CAT activity by 13% in plants exposed to 50 mM salt. BC supplementation also enhanced POD activity by 19%, 20%, and 24% in plants exposed to 50, 100, and 150 mM salt, respectively, compared to the corresponding salt-stressed but unsupplemented plants. CHT supplementation improved the POD activity by 16% and 20% in plants exposed to 50 and 100 mM salt stress compared to the corresponding salt-stressed but unsupplemented plants. BC supplementation increased the activity of GST by 14%, 21%, and 25% and GPX by 16%, 22%, and 26% in plants exposed to 50, 100, and 150 mM NaCl, respectively, compared to the corresponding unsupplemented salt-stressed plants. CHT supplementation increased GST activity by 14%, 16%, and 20% and GPX activity by 13%, 15%, and 14% in plants treated with 50, 100, and 150 mM NaCl, respectively, compared to the corresponding salt-stressed but unsupplemented plants (Figure 7A–E).

### 3.7. Methylglyoxal Detoxification System

**Figure 8:**
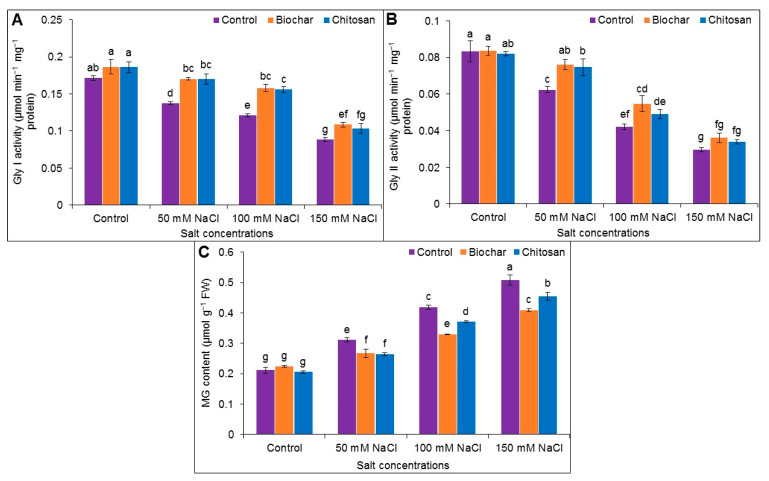
Activities of Gly I (**A**), Gly II (**B**), and MG content (**C**) of jute as affected by biochar and chitosan under different concentrations of NaCl. Mean value (±SD) was calculated from three replications, and different letters on the columns indicate significant differences among the treatments at *p* ≤ 0.05 applying Fisher’s LSD test.

The MG content notably increased, whereas the activity of Gly I and Gly II decreased in salt-stressed plants compared to the untreated control plants. BC and CHT supplementation decreased the MG content in salt-stressed plants. BC supplementation increased the activity of Gly I (by 19%, 23%, and 18%) and Gly II (by 18%, 22%, and 18%) in plants exposed to 50, 100, and 150 mM salt, respectively, while CHT supplementation enhanced the activity of Gly I by 19%, 22%, and 15% and Gly II by 16%, 14%, and 13%, respectively, compared to the corresponding salt-stressed but unsupplemented plants (Figure 8A–C).

### 3.8. Na^+^ and K^+^ Content

**Figure 9:**
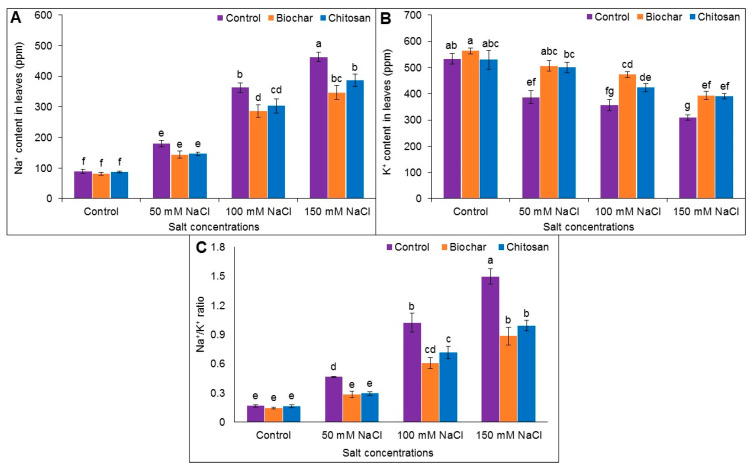
Na^+^ content (**A**), K^+^ content (**B**), and Na^+^/K^+^ ratio (**C**) of jute as affected by biochar and chitosan under different concentrations of NaCl. Mean value (±SD) was calculated from three replications, and different letters on the columns indicate significant differences among the treatments at *p* ≤ 0.05 applying Fisher’s LSD test.

Salt stress significantly increased the Na^+^ content and reduced the K^+^ content in the leaves of jute plants. Pronounced accumulation of Na^+^ content (by 81%) and reduction of K^+^ content (by 72%) were observed in plants treated with 150 mM NaCl compared to the untreated control plants. Similarly, the Na^+^ content increased by 51% and 75% in plants treated with 50 mM and 100 mM salt, while the K^+^ content decreased by 50% and 72%, respectively, compared to the untreated control plants. However, the reduction in Na^+^ content and the increase in K^+^ content resulted in an increase in the Na^+^/K^+^ ratio in the BC- and CHT-supplemented plants compared to the salt-stressed but unsupplemented plants (Figure 9A–C).

### 3.9. Phenotypic Appearance

**Figure 10:**
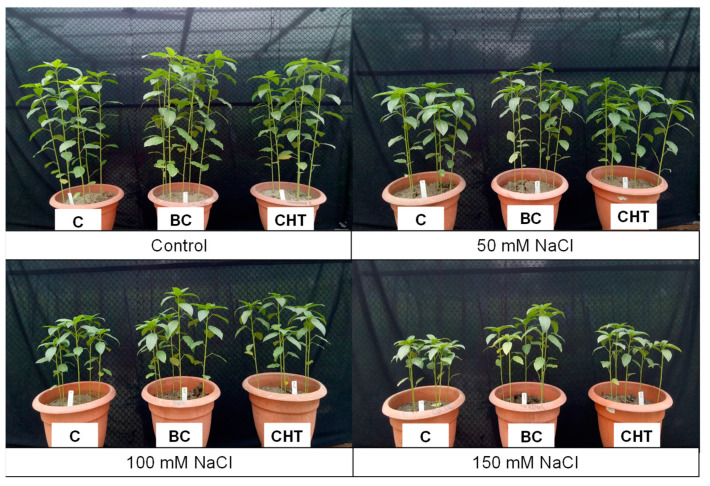
Phenotypic appearance of jute plants as affected by biochar and chitosan supplementation at different concentrations of NaCl.

The phenotypic appearance of salt-stressed jute plants with/without BC or CHT supplementation is shown in Figure 10. The growth of salt-treated plants was retarded due to salt stress but was improved by BC and CHT supplementation compared to the corresponding salt-stressed but unsupplemented plants (Figure 10).

## 4. Discussion

Salt stress decreases the hydraulic conductivity of plant cells due to the excessive amount of soluble and exchangeable ions in the soil solution. Thus, water adsorption capacity and the influx of water into cells decreases, thereby hampering cell expansion and reducing plant growth [42]. Chlorophyll, which is responsible for net photosynthesis activity, also declines in level during salt stress, causing lower photosynthetic assimilate production and biomass accumulation by salt-stressed plants [43]. In the present study, growth in terms of length, FW, and DW of both roots and shoots, as well as in stem diameter, were decreased in the salt-stressed jute plants, in agreement with previous findings by Naik et al. [44] and Rahman et al. [45]. However, BC and CHT applications helped to mitigate the salt stress effects and improved jute plant growth.

The uptake of essential mineral nutrients and plant growth are curtailed due to salt stress, but both processes were improved by BC supplementation [46]. BC amendment improves the soil physio-chemical properties by adsorbing excess Na^+^ ions under saline conditions and improves plant growth by maintaining cell proliferation and expansion by maintaining water potential [47]. On the contrary, CHT application has been shown to improve the growth and photosynthetic activity in cadmium (Cd)-stressed pea plants by forming complexes with Cd and enhancing essential nutrient uptake [48]. Mehmood et al. [11] also observed enhanced plant growth and biomass accumulation when BC was applied, either alone or in combination with CHT, to soybean plants growing under saline conditions.

One of the earliest effects of salt stress is physiological drought, as the presence of high concentrations of Na^+^ and Cl^−^ ions increases the osmolarity of soil solution and reduces the water uptake capacity of plants [49]. Upon exposure to salt stress, the RWC of the jute plants in the present study was reduced; however, the plants mitigated the adverse effects of salt-induced dehydration by reducing transpiration and accumulating osmoprotectants, such as Pro [10]. The accumulation of Pro showed dose-dependent increases in response to NaCl stress, but the Pro levels were reduced in BC-amended soil in the present study. Supplementation with BC resulted in a higher water use efficiency and an improved leaf water content, thereby helping to mitigate osmotic stress and lessen the need for osmolyte (Pro) accumulation [50,51]. Similar results were also observed with CHT application in water-stressed barley, where Pro accumulation was reduced by regulating the water pressure and RWC [20]. Tanzeem-ul-Haq et al. [52] reported that combined CHT and BC supplementation enhanced the RWC of lentil plants in nickel (Ni)-spiked soils and improved plant growth by improving moisture retention and plant—water relationships.

Salt stress exerts its negative effects on plant physiology and metabolic processes by inducing osmotic stress, ionic toxicity, and water and nutrient imbalances and by accelerating ROS accumulation, which collectively threatens the antioxidant defense mechanisms of plants [4]. A certain level of ROS accumulation is beneficial for plant growth and redox regulation and for transporting signals to different cellular compartments through the mitogen-activated protein kinase (MAPK) pathway. However, excess generation of ROS destroys cellular components, including lipids, proteins, DNA, and carbohydrates, and triggers programmed cell death [53,54]. In the present study, the H_2_O_2_ content increased with increasing salt concentrations. Higher accumulation of H_2_O_2_ results in accelerated lipid peroxidation (causing an increase in MDA content) and LOX activity (associated with lipid disintegration), and all these responses increase the EL under salt stress. The present findings agree with the previous studies by Hasanuzzaman et al. [55], Siddiqui et al. [56], Rahman et al. [23], and Parvin et al. [57]. Reductions in MDA, H_2_O_2_, LOX activity, and EL with BC amendment have been reported in maize varieties by Alharby et al. [47]. Similarly, salt-stressed tomato plants showed reduced H_2_O_2_ and MDA contents in response to CHT treatment [58]. AlKahtani et al. [59] reported that the generation of MDA, H_2_O_2_, and EL declined with the application of CHT in salt-stressed sweet peppers. Amendment with BC, CHT, or their combination decreased the MDA and H_2_O_2_ contents and protected lentil plants from Ni-induced oxidative damage [52].

The non-enzymatic antioxidants AsA and GSH play pivotal roles in scavenging ROS through the AsA-GSH cycle, which helps to protect cellular organelles and biomolecules from oxidative damage. AsA is a strong antioxidant and electron donor, so it neutralizes ROS by delocalization of electrons, and it also participates in the biosynthetic pathway of some phytohormones. In contrast, GSH is a non-protein thiol compound that scavenges ROS through the AsA-GSH cycle and maintains redox homeostasis [1]. Salt stress decreased the contents of both AsA and GSH in the jute plants, whereas DHA and GSSG contents increased with increasing salt concentrations. The end effect was a reduction in the AsA/DHA and GSH/GSSG ratios in the current study. Similar results were reported by previous researchers [7,55,57]. Application of BC and CHT further increased the content of AsA and GSH and reduced the content of DHA and GSSG, thereby improving the capability for ROS detoxification under salt stress. The result was an upregulation of the AsA/DHA and GSH/GSSG ratios and maintenance of redox balance. BC supplementation was also reported to increase the AsA content in mung bean plants grown in Ni-contaminated soil [60].

Four key enzymes—APX, MDHAR, DHAR, and GR—are directly involved in the cycle that links the AsA-GSH cycle for scavenging ROS (H_2_O_2_) and for regenerating AsA and GSH. In the AsA-GSH pool, APX participates in scavenging H_2_O_2_ by producing monodehydroascorbate (MDHA), which is transformed into AsA in the presence of MDHAR. In another non-enzymatic reaction, MDHAR is reduced into DHA to regenerate AsA in the presence of DHAR (a GSH-dependent enzyme). The reduced form of GSH, which was previously oxidized to GSSG, is then regenerated through the action of GR [61]. In the present study, salt stress increased APX activity (in response to up to 100 mM NaCl) and was also responsible for reductions in the MDHAR, DHAR, and GR activities. These results also agree with previous findings [7,23]. For example, APX activity was improved with BC amendment in Cd-stressed quinoa plants [50], in Ni-stressed mung bean plants [60], and in drought-stressed maize plants [47]. The activities of MDHAR and DHAR were also augmented by BC addition in maize plants under water deficit [47]. The activity of DHAR was also enhanced in mung bean plants under Ni-induced oxidative stress [60], and similar results were also reported by Haider et al. [62] in pea in lead (Pb)-stressed plants. Increased APX activity was also reported in salt-treated soybean plants following BC and CHT supplementation [11], in agreement with the present findings. Supplementation with BC and/or CHT also enhanced APX activity in lentil plants under Ni stress [52]. Augmentation of GR activity was also reported in response to BC and CHT supplementation in barley under drought stress [20]. In the present study, BC and CHT supplementation improved APX, MDHAR, DHAR, and GR activities in jute plants exposed to salt stress.

The frontline of defense in scavenging ROS in plants is the SOD enzyme, a metalloenzyme that catalyzes a dismutation of O_2_^•−^ into H_2_O_2_, thereby reducing the generation of hydroxyl radicals (^•^OH). Subsequent conversion of H_2_O_2_ to H_2_O and O_2_ depends on the enzyme CAT [63,64]. In the current study, SOD activity was enhanced but CAT activity was reduced in salt-stressed jute plants, in agreement with previous reports [45,57]. Glutathione peroxidase (GPX), which belongs to the non-heme POD family, utilizes GSH and thioredoxin to scavenge H_2_O_2_ and other hydroperoxides. Other xenobiotics and electrophilic substances are also degraded into less toxic compounds by the action of the GST and GPX enzymes and are ultimately sequestered in vacuoles [1]. The activities of SOD, POD, and CAT were augmented by the application of BC under salt stress [11]. Sofy et al. [51] also reported that BC amendment increased the activities of SOD, CAT, and POD to regulate the accumulation of ROS under salt stress. Detoxification of H_2_O_2_ by enhancing CAT and POD activity with CHT amendment resulted in increased tolerance of milk thistle plants to salinity [65]. Foliar application of low- and medium-molecular-weight CHT to durum wheat under salt stress increased the activities of SOD and CAT [66]. Likewise, SOD, CAT, and POD activities were increased following foliar application of CHT in salt-stressed tomato plants [58]. Notable increases in CAT, SOD, and POD activities and increased ROS scavenging were reported following BC addition in drought-stressed maize varieties [47]. A profound increase in SOD and CAT activity by BC and CHT application was reported in Ni-stressed lentil plants, confirming enhanced ROS scavenging to protect the plants against oxidative damage [52]. Similarly, the application of BC increased SOD and CAT activities in Ni-stressed mung bean plants [60]. The activities of SOD, POD, and CAT were also increased in Cd-stressed quinoa plants following BC supplementation [50].

Methylglyoxal is a cytotoxic compound that increases greatly in level in response to environmental stresses. Plants are protected from MG-induced cellular damage by the action of glyoxalase enzymes (Gly I and Gly II), which play a crucial role in the detoxification of MG by a two-step reaction. MG is first converted into SLG via GSH in the presence of the Gly I enzyme, and then D-lactic acid is produced from SLG through the enzymatic action of Gly II [55]. In the present study, the MG content increased, whereas the Gly I and Gly II activities declined under salt-stress conditions. These results agree with previous research findings [23,43,57]. The application of BC increased the Gly I and Gly II activity in the salt-stressed plants, thereby reducing MG-induced cellular damage. Similarly, CHT also reduced MG toxicity by promoting Gly I and Gly II enzyme activity in the salt-affected jute plants in the current study.

Salt stress increases Na^+^ and Cl^−^ concentrations across the root zone and disrupts the ionic homeostasis in plants, while also reducing water and nutrient uptake capacity from the soil solution. The presence of increased amounts of soluble ions in the soil medium increases the transportation of Na^+^ ions to various parts of the plants. Accumulation of Na^+^ ions decreased the K^+^ content in the jute plants in the current study, and K is considered an essential nutrient for carrying out different metabolic activities. The results presented here agree with other findings in plants under salt stress [7,45,57]. Soil supplemented with BC adsorbed the excess soluble ions (Na^+^ and Cl^−^) and increased K^+^ uptake, thereby helping to maintain ion homeostasis under salt stress. Similarly, CHT can also bind Na^+^ and Cl^−^ ions and improve K^+^ mobility in plants. Yang et al. [67] demonstrated that BC supplementation decreased Na^+^ content and increased K^+^ availability in quinoa under saline conditions. Similarly, a reduced Na^+^ accumulation has been observed following the application of BC and CHT under salt stress conditions in soybean [11]. Hafez et al. [12] mentioned that soil amendment with BC reduced Na^+^ and augmented K^+^ uptake, thereby reducing ion toxicity by balancing the Na^+^/K^+^ ratio in salt-stressed wheat plants.

**Figure 11:**
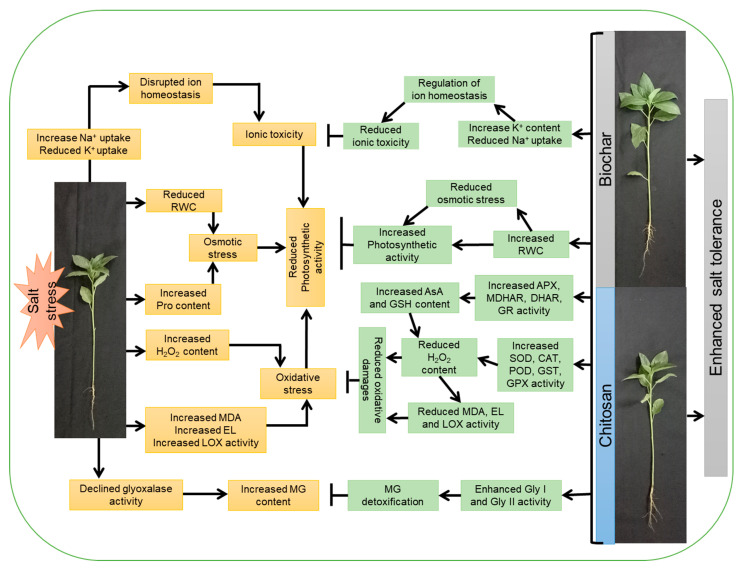
A possible mechanism for improvement in salt tolerance in jute by biochar and chitosan amendments based on the findings of the current study.

In summary, exposure of jute plants to salt stress decreased plant growth, biomass accumulation, stem diameter, and photosynthetic pigment contents, but these decreases were mitigated by the exogenous supplementation of BC and CHT. The RWC decreased, whereas the Pro content increased, to protect the plants from the osmotic stress created by saline conditions. Oxidative stress indicators (H_2_O_2_ and MDA content), EL, and LOX activity were increased with increasing NaCl concentrations. Amendment with BC and CHT increased the RWC and reduced the Pro content, while also reducing the oxidative damage by decreasing the MDA and H_2_O_2_ content and the EL and LOX activity in salt-stressed jute plants. Salt stress decreased the AsA and GSH content, but increased the DHA and GSSG content, thereby reducing the AsA/DHA and GSH/GSSG ratios. However, BC and CHT supplementation enhanced the accumulation of AsA and GSH, followed by decreases in the content of DHA and GSSG, resulting in increased AsA/DHA and GSH/GSSG ratios. Salt stress decreased the MDHAR, DHAR, GR, CAT, POD, GST, and GPX activities in jute plants, but increased the APX (up to 100 mM NaCl stress) and SOD activities. Amendment with BC and CHT improved the defense responses of jute plants by enhancing antioxidant enzyme activity. MG accumulation increased and Gly I and Gly II activity decreased in response to salt stress, whereas BC and CHT supplementation enhanced Gly I and Gly II activity to support MG detoxification. The Na^+^ ion content increased under salt stress and reduced K^+^ accumulation. However, BC and CHT amendment decreased the Na^+^ accumulation and enabled K^+^ uptake under salt stress. Taken together, the results obtained in the current study confirm that both BC and CHT are capable of mitigating salt-induced oxidative stress by modulating the ROS metabolism, antioxidant defense system, and glyoxalase system to improve plant growth and physiology under salt stress (Figure 11).

## 5. Conclusions

The present investigation evaluated the role of BC and CHT as protectants from salt-induced damage in jute plants. Under salt stress, the jute plants showed responses indicative of ionic and osmotic stress, ROS over-accumulation, lipid peroxidation, enhanced LOX activity, MG accumulation, growth reduction, and impaired physiobiological activities. Supplementation with BC and CHT enhanced the activities of both non-enzymatic and enzymatic antioxidants to mitigate ROS-induced damage. Supplementation with BC and CHT decreased MG toxicity by upregulating Gly I and Gly II activity while also maintaining ion homeostasis and osmotic balance. Overall, the SPAD value, RWC, growth parameters, and phenotype appearance were improved by BC and CHT supplementation under salt stress. However, further investigations should be carried out to explore the actual mechanisms by which BC and CHT perform protective roles and to elucidate the complex interaction between the different biochemical pathways involved in salt stress tolerance.

## Data Availability

All data are available in this article.
